# RNAi-Based Biocontrol Products: Market Status, Regulatory Aspects, and Risk Assessment

**DOI:** 10.3389/finsc.2021.818037

**Published:** 2022-01-05

**Authors:** Kristof De Schutter, Clauvis Nji Tizi Taning, Lenny Van Daele, Els J. M. Van Damme, Peter Dubruel, Guy Smagghe

**Affiliations:** ^1^Laboratory of Agrozoology, Department of Plants and Crops, Faculty of Bioscience Engineering, Ghent University, Ghent, Belgium; ^2^Polymer Chemistry and Biomaterials Group, Department of Organic and Macromolecular Chemistry, Center of Macromolecular Chemistry, Faculty of Sciences, Ghent University, Ghent, Belgium; ^3^Laboratory of Biochemistry and Glycobiology, Department of Biotechnology, Faculty of Bioscience Engineering, Ghent University, Ghent, Belgium

**Keywords:** RNAi, RNA-based pesticides, spray-induced gene silencing (SIGS), regulatory framework, risk assessment, plant protection

## Introduction

Climate change and the increasing demand for food by the growing population present enormous challenges for food security. With a population that will likely grow from 7.5 billion people today, to nearly 10 billion by 2050, the food demand will drastically increase while the available area for agriculture cannot increase without endangering biodiversity. This takes into account that agriculture already occupies nearly 40% of the earth's surface and irrigation of agricultural crops comprises 70% of global water use (1). Climate change will increase abiotic stresses (drought, heat, flooding, salinity, etc.), causing a reduction in cultivable land and crop yield losses (ranging from 50 to 70%) (2). In addition, it will also increase biotic stresses, including a 10–25% increase expected in insect damage per global temperature degree increment in the next years (3). Farmers rely mainly on the use of synthetic pesticides and herbicides to protect crops from these biological stresses. However, the excessive use of these conventional chemicals in the last decades has had serious detrimental effects on the environment and has also promoted the emergence of resistance in pest populations. Consequently, there is a pressing need for alternative, selective, environmentally friendly, and sustainable solutions for pest control and crop improvement.

In this context, RNA interference (RNAi)-based biocontrol has emerged as a good alternative to hazardous pesticides (4). Due to the possibility to design the active molecule (double-stranded RNA, dsRNA) to be species-selective and its biodegradability in the environment, RNAi-based biocontrol is considered to have a low environmental impact (5). RNA interference is a natural regulatory and defense mechanism present in most eukaryotic organisms. The presence of free dsRNA in the cell triggers RNAi and directs sequence-specific degradation of messenger RNA (mRNA) molecules resulting in effective gene silencing. While in nature, RNAi is best known as a defense mechanism against viruses, RNAi technology can also be utilized by scientists to turn off the expression of individual genes to study their function. Additionally, the RNAi mechanism can be exploited as a species-selective pest control strategy through silencing of essential genes required for the pest's survival. RNA interference applications have been developed in the form of genetically modified (GM) plants expressing dsRNAs that either silence essential genes in pests or pathogens or target endogenous plant genes to improve plant qualities, and is termed as host induced gene silencing (HIGS). Alternatively, RNAi-based products can also be applied exogenously through spray application, further referred to as spray induced gene silencing (SIGS) (4). The exploitation of RNAi to improve plant health is a fast-growing market and while GM RNAi plants are being assessed using the existing regulatory framework, appropriate safety evaluations, and authorization procedures for SIGS-based products are less clear.

## Moving to the Market

Over the last two decades, the screening and functional analysis of potential target genes and the design of RNAi-based strategies for crop protection and crop improvement, has led to the first commercial RNAi-based products entering the market. The first product of this kind was approved by US regulators in 2017 and recently also by the Chinese regulators in 2021. The Bayer “SmartStax Pro” maize (Mon87411) combines the expression of the *Bacillus thuringiensis* (Bt) Cry3Bt1 toxin with glyphosphate resistance and the expression of a dsRNA targeting the *Snf7* gene of the western corn rootworm (*Diabrotica virgifera virgifera*). RNA interference-mediated silencing of this gene involved in the transport of transmembrane proteins causes lethality in *D. v. virgifera*, ultimately leading to reduced root damage (6). The combination with the *Bt*-toxin improves target pest control and resistance management (7). This product will be available to farmers in the US in 2022 and from 2023 in Canada. In Europe, the “SmartStax Pro” maize has been authorized for the market for all uses except cultivation (8).

Host induced gene silencing is not limited to pest control and several products using RNAi to improve crop quality have already been authorized for commercialization or will reach the market in the near future. For example, The Bayer “Vistive gold” high-oleic soybeans (Mon87705), in which a gene in the fatty acid biosynthesis pathway is targeted, has been approved for food, feed, and cultivation in the USA, Canada, and Japan, and for feed and food use in the EU market (9). In 2023, the Australian scientific agency, CSIRO (Commonwealth Scientific and Industrial Research Organization) and the Australian clean technology business, GO Resources, are expected to release “Super-High Oleic” (SHO) safflower in Australia. Commonwealth Scientific and Industrial Research Organization's gene silencing technology is designed to switch off target genes in developing seeds, thereby causing an accumulation of oleic acid content in the seeds without compromising plant performance. In these seeds, the enzymes required for the conversion of oleic acid into polyunsaturated fatty acids are silenced during seed oil synthesis, attaining a higher value for the biofuel, chemical, and lubricant industry. Recently, the USDA deregulated Simplot GM “Innate” potatoes. In these potatoes, RNAi-mediated silencing prevents potato bruising, reduces acrylamide production, and improves starch quality. In “HarvXtra” alfalfa from Forrage genetics, the lignin content is reduced by RNAi-mediated silencing, making the crop more digestible for cattle. In the future, applications for crop improvement are expected to increase as RNAi has been demonstrated to be a powerful tool to enhance crop quality and performance, including the development of seedless fruits, plant biomass regulation, flower coloration, scent development, shelf-life enhancement, secondary metabolite regulation, and abiotic stress tolerance (10–12).

Due to the negative public perception of GMOs, the technical challenges involved in the transformation of many crop species and the time and cost for obtaining regulatory approval, scientists and companies are exploring non-GMO based approaches through which dsRNA can be exogenously applied, i.e., the SIGS-approach (5). This approach allows high targeted plant protection against pests and diseases without the need for plant transformation. An insect receiving considerable focus for a SIGS-based commercial exploitation, due to its high sensitivity to RNAi effects, is the Colorado potato beetle, *Leptinotarsa decemlineata*. Recently, Syngenta and Greenlight Biosciences have successfully concluded independent field trials showing a better performance of dsRNA-treated plants to control the Colorado potato beetle (13, 14). In addition to SIGS-based plant protection products, RNAi-based products for the protection of pollinators are moving to the market. Field tests under “BioDirect,” a SIGS-based platform developed by Bayer, has confirmed the potential of RNAi technology to control *Varroa destructor* mites in honeybees, by reducing mite levels and increasing colony survival rates (15).

With SIGS-based products moving to the market, there is a need for a clear regulatory framework and defined guidelines for risk assessment and registration of these novel plant protection products.

## Regulatory Framework in USA, Europe, and Australia

### USA

**Figure 1:**
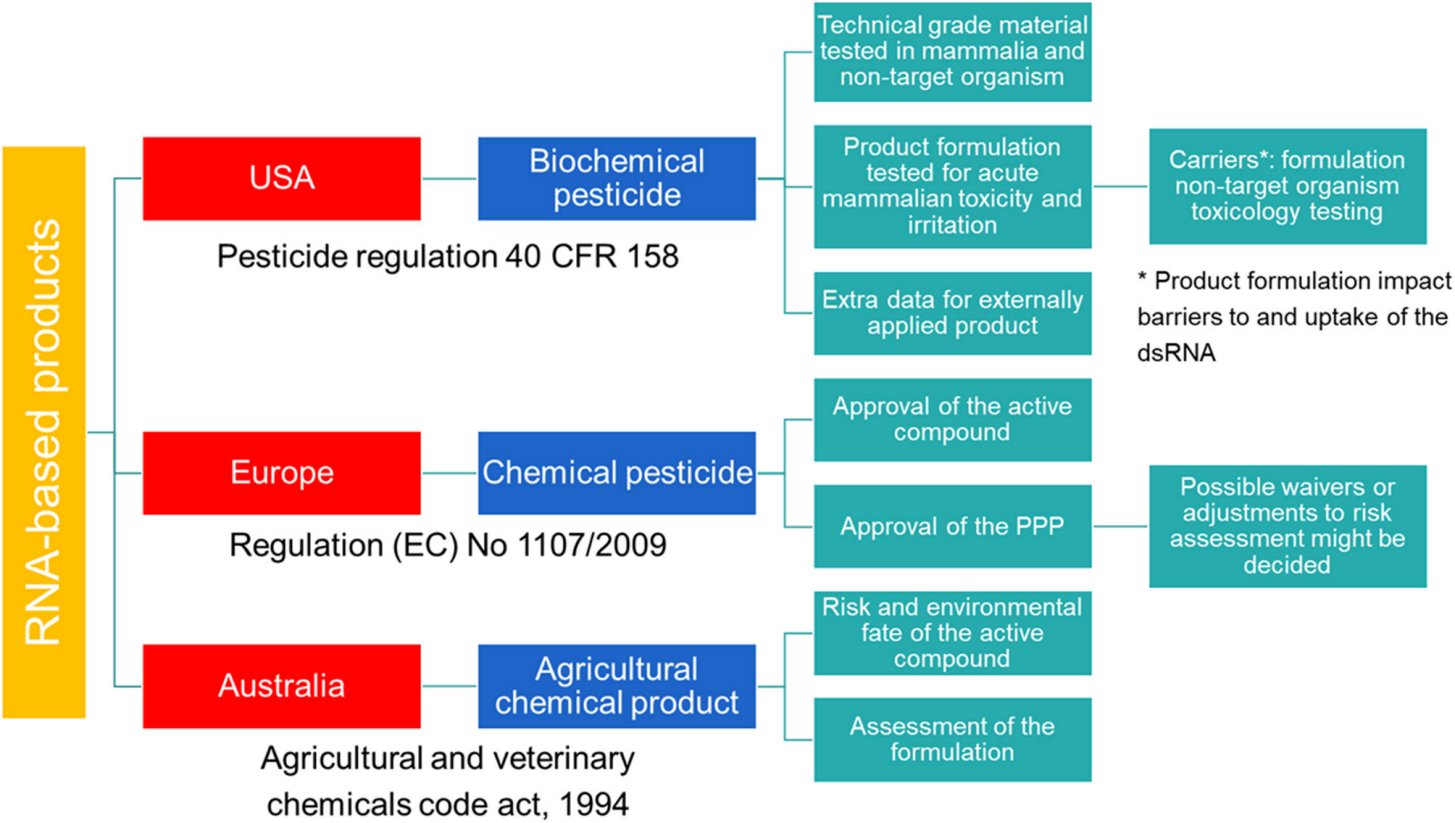
Schematic overview of the regulatory frameworks for RNA-based products in the USA, Australia and Europe.

In the USA, SIGS-based RNAi products for plant protection are considered biochemical pesticides (Figure 1). In contrast to conventional pesticides, which are generally synthetic compounds that directly kill or inactivate the pest, biochemical pesticides are naturally occurring compounds which are usually inherently less toxic (16). However, these biochemical pesticides still require a US Environmental Protection Agency (EPA) registration before manufacture, transport, and sale (17). Environmental Protection Agency provides approval under the Federal Insecticide, Fungicide, and Rodenticide Act (FIFRA) and the Federal Food, Drug, and Cosmetic Act (FFDCA), and bases the approval on a risk/benefit standard. Therefore, no unreasonable adverse effect(s) to man or the environment should result from the use of the pesticide in order to support its registration under FIFRA. In addition, FFDCA allows to set maximum residue levels for pesticides used in or on food or feed (16). During this investigation, technical grade material of the pesticide is tested along with product formulation. For field testing prior to registration, an experimental use permit is required.

While no specific data requirements for sprayable or externally applied dsRNA-based pesticides are available, the EPA requires additional data as described in Title 40 of the Code of Federal regulations (CFR) Part 158 for biochemical pesticides. Next to the requirements for conventional pesticides, these articles list requirements for biochemical pesticides under subpart U, and request that the active ingredient as well as the final product must be evaluated. Although, some of the data could be appropriate to be waived according to 40 CFR 158.45, specific data for sprayable dsRNA-based products are required. Additional data might also be required when using formulations that impact RNAi barriers, including product-specific formulation as well as non-target organism toxicology testing to better assess the potential for hazard.

Spray-induced gene silencing-based products are not considered GMOs, unless they would contain viable GM organisms, e.g., engineered bacteria producing dsRNA. When genetically engineered organisms are used to produce the dsRNA, while being non-viable in the final product, they are considered a pesticide intermediate under the Toxic Substances Control Act (TSCA) and require submission of a Microbial Commercial Activity Notice (MCAN) before initiating manufacture (18).

### Australia

Australia has a modern and efficient agricultural industry, with focus on the development of innovative systems through research and design to improve food production and sustainability (19). In the context of RNAi-based products, Australia has played a pioneering role in providing a legal structure for the approval of these crop protection products. As of the 8^th^ of October 2019, dsRNA-based products applied topically to protect plants against insects, fungal, and viral pests are defined as agricultural chemical products (Figure 1). Previously, these products were regulated by the office of the gene technology regulator (OGTR) and the Australian Pesticides and Veterinary Medicines Authority (APVMA). However, the OGTR's “Technical Review of the Gene Technology Regulations 2001” suggested that techniques involving the application of RNA to an organism to temporarily induce RNAi do not constitute gene technology and that the resulting organisms are not considered GMO. Therefore, SIGS-applications are not subjected to the OGTR regulation and should be regulated in a manner proportional to the risk they pose. However, this is on the condition that the RNA cannot be translated into a polypeptide, the organism's genomic sequence cannot be altered and no infectious agent is produced.

While there are currently no specific guidelines for data requirements in support of the registration of RNAi-based products in agriculture, at a minimum, data on the chemistry and manufacture, human health, worker health and safety, environmental fate and toxicity, efficacy, and crop safety data are required. To help prospective applicants in the registration process, the APVMA provides pre-application support.

### Europe

In the European Union (EU), HIGS based products are regulated as GMOs and fall under the scope of Directive (EC) 2001/18 (20). However, when these plants are intended for food and feed products, they fall under the Regulation (EC) 1829/2003 (21). An important aspect related to SIGS-based products will be whether they contain living organisms or only purified molecules (22). When these products contain viable GM organisms, they must be authorized according to EC Directive 2001/18 (20). In contrast, when no GM organisms have been used or it is guaranteed they have all been inactivated, the SIGS-based products are not considered GMOs. For these SIGS-based products, no specific category is available and registration of these RNAi-based products follows the same regulatory framework as for the classical synthetic pesticides (Figure 1). The legal basis for the authorization of all pesticides to protect crops or other useful plants, or plant protection products (PPPs), is provided by Regulation (EC) 1107/2009 (23) and comprises a two-step approach. First, the active compound is assessed by the European Food Safety Authority (EFSA) and approved by the EU Commission. Second, the PPP containing the active compound is evaluated. While the risk assessment of the active compound is EU-wide, the authorization of the PPPs is decided by the member states (MS). To simplify the authorization of the PPP, a zonal approach was introduced by Regulation (EC) 1107/2009 (23). Within one zone (Northern, Central, and Southern zone), authorization by one MS, the zonal rapporteur MS, is sufficient for the whole zone. However, individual MS can still make claims on national ecological or agricultural specificities and decide on specific risk management options for their country.

Double-stranded RNA can be considered a new class of active substances and must be assessed according to the data requirements for active substances described in Regulation (EC) 283/2013 (24). Specific data requirements for SIGS-based PPPs have not been specified and assessment is performed using the same requirements for chemical PPPs as described in Regulation (EC) 284/2013 (25) and Regulation (EC) 546/2011 (26). However, possible waivers for specific areas of concern can be decided on a case-to-case basis. Additionally, guidance documents by the Organization for Economic Co-operation and Development (OECD), the European and Mediterranean Plant Protection Organization (EPPO) and EFSA provide detailed support on the methodological requirements for the risk assessment of active substances and PPPs (22). RNA-based PPPs have different properties compared to the chemicals used as active substances in current PPPs. Therefore, adaptations of the data requirements are reasonable and new assessments and tools must be introduced, e.g., bioinformatics for the determination of off-target effects (22). It is therefore included in article 77 of the Regulation (EC) 1107/2009 that the Commission may adopt or amend technical and other guidance documents for the implementation of this regulation (23). Such specific adaptations have already been implemented for microorganisms, pheromones and botanicals. For dsRNA-based PPPs, first considerations and recommendations have been presented by the OECD (27). However, it might still take some time before these rules are implemented.

Within the EU, there is a drive to replace contentious pesticides and agrochemical inputs such as pesticides, fertilizers, and antimicrobials with safe, efficient, and cost-effective alternatives to ensure sustainable food production. Within this European Green Deal, the unique specificity and efficacy of the RNAi-based PPPs suggests them as promising solutions to substitute conventional pesticides (28). However, with SIGS-based PPPs in the pipeline, there is a need to discuss regulatory and biosafety issues in order to better define an appropriate framework and risk assessment procedure for these products.

## Risk Assessment

Being natural molecules involved in gene regulation and virus defense, dsRNA molecules are widespread in plants and animals. Consequently, they are natural components of food and feed (29) and have, therefore, a long history of safe consumption by humans and other vertebrates (27). It has been suggested that the widespread presence of RNAs in the environment and food have resulted in the effective physiological and biochemical barriers against RNA observed in mammals (27). Due to enzymatic degradation and cellular uptake barriers present in the gut of humans and farm animals, exposure through ingested dsRNAs is considered negligible (30, 31). In addition, there is no scientific basis to suggest that small dsRNAs present in HIGS GM foods have different properties or pose a greater risk than those already naturally abundant in conventional foods (32). Despite the inherent low hazard of the active molecule, all technologies carry a set of potential risks (5). However, careful analysis of these risks can allow the design of safe use practices to limit or mitigate potential detrimental effects (5).

**Figure 2:**
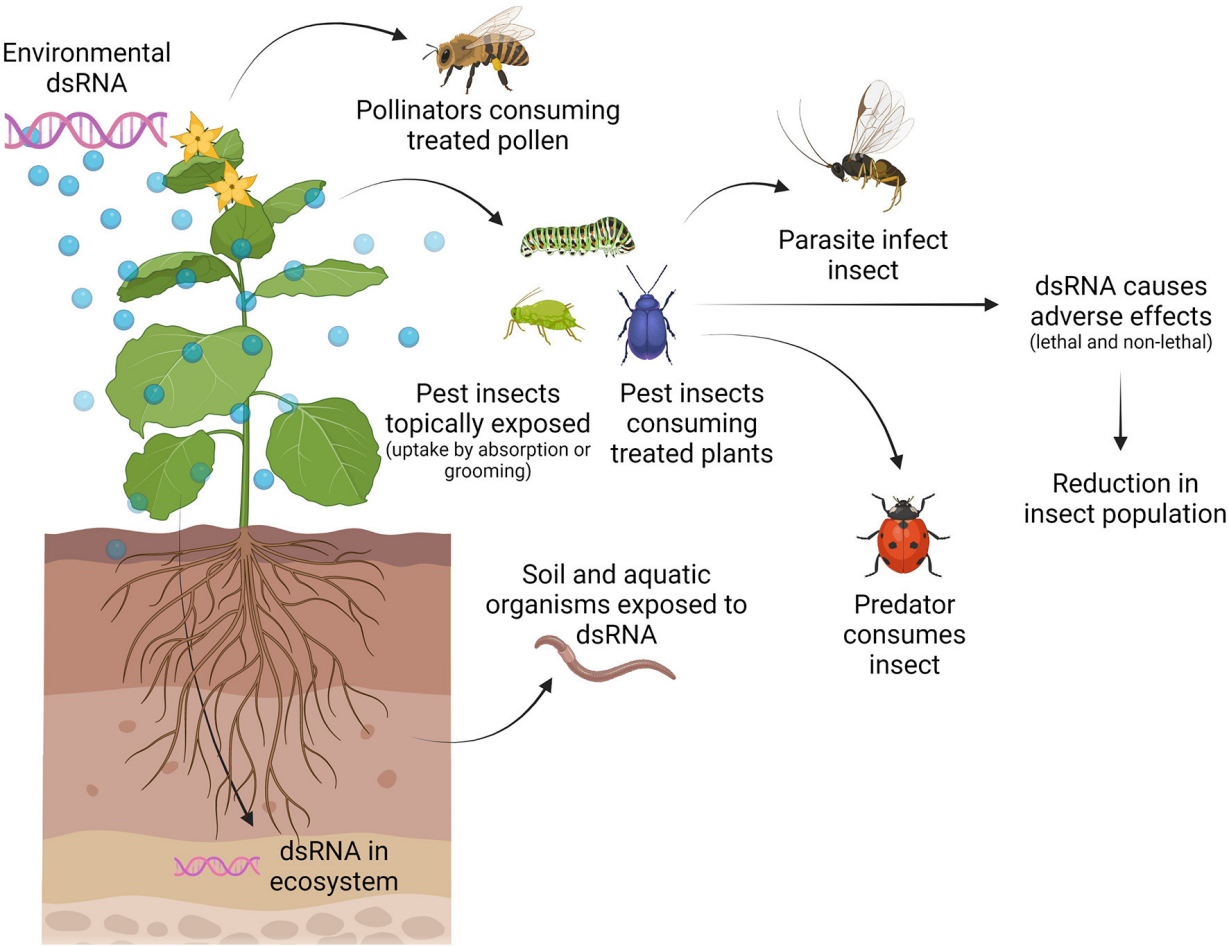
Exposure pathways of target and non-target organisms to externally applied dsRNA. The application of dsRNA leads to the presence of dsRNA on/in the plant and the ecosystem. Target (pest) and non-target (such as pollinators) insects can get exposed by direct contact with dsRNA on/in the plant (feeding and/or grooming) or during dsRNA application (topically). Furthermore, natural enemies (predators and parasitoids) of the pest can get exposed to the dsRNA while feeding on the pest. Other beneficial organisms such as soil decomposers and aquatic organisms can get exposed to dsRNA in the ecosystem. Figure is created with BioRender.com.

We must understand how and when organisms can be exposed to applied dsRNA and how they can get harmed, as this will allow us to define testable risk hypotheses (7). For example, when applying RNAi-based products in the field against insect pests, the target pest will be exposed to the dsRNA molecules, but in addition, also non-target organisms as well as the environment might be exposed (Figure 2). Target and non-target species can be exposed directly to dsRNA during feeding on treated plants or through absorption or grooming after topical application or through contact with dsRNA in the environment (i.e., dsRNA present in soil or water) (Figure 2). Furthermore, natural enemies might get exposed to dsRNA by feeding on a pest that has been exposed to dsRNA (Figure 2) (33). The exposure of non-target organisms is dependent on several parameters, including application rate, timing of application, application method, number of applications, off-site movement of the dsRNA, and stability and persistence of the dsRNA (7). The stability of the active component, the dsRNA, in the environment is very low. Microbial nucleases present in the soil and on leaves, UV-radiation, and run-off due to dew and rain can significantly limit the availability of dsRNA to the pest (34, 35). Therefore, stabilizing formulations are required to allow the successful usage of dsRNA in a SIGS approach. Formulation technologies can also be used to improve cellular internalization of dsRNA and to protect dsRNA against nucleolytic degradation, hence improving overall delivery to the pest (36–38). Since the use of formulations could impact the persistence of dsRNA in the environment and the human exposure pathways, formulations will need to be taken into account in the risk of exposure and may require assessment on a case-to-case basis (35). In addition, formulations might present a risk of their own and the impact of the formulation itself on the environment and non-target organisms should be assessed as well.

Double-stranded RNA can pose a hazard to non-target species in a sequence-specific or a sequence-unspecific manner. Although RNAi is inherently highly specific for a target organism or group of organisms, partial homology of the dsRNA can cause potential effects in a non-target species (39). Bioinformatics could assist in identifying off-target sequences and potential effects in non-target species. However, as indicated in the EFSA literature review on environmental risk assessment (ERA), certain knowledge gaps impede these bioinformatic predictions (39). These gaps are related to fundamental insight into the RNAi mechanism, e.g., the differences between organisms in the processing of dsRNA and the stringency of base pair mismatches. For example, it was reported that siRNAs, which are the result of Dicer-2 processing, can have a variable length (20–22 nucleotides) in different insect species or insect orders (40). Furthermore, while the miRNA pathway will allow several mismatches to still have efficient silencing, this knowledge is fragmentary and even contradictory for the siRNA pathway (39). In addition, it was observed that the position and the type of the mismatch between the siRNA and target mRNA play a crucial role in the efficacy of the silencing (41). Therefore, bioinformatics can only be one component of the risk assessment. While it can help in the selection and prioritization of non-target species, it should be complemented by an empirical approach where a range of organisms, closely related and phylogenetically more distant, are exposed to the dsRNA and their responses analyzed (7). At the same time, bioinformatics tools should be augmented through continuous input from fundamental research on the RNAi machinery, genomic data libraries, and improved algorithms.

Next to these sequence-specific effects, organisms can respond to dsRNA in a manner independent of the sequence of the dsRNA, the sequence-unspecific effects. These include saturation of the RNAi pathway and activation of the defense or immune pathways. When dsRNA is administered at high concentrations, it could theoretically saturate the core RNAi machinery (42). Since RNAi is a part of the antiviral immunity of the cell, such a saturation could have a substantial impact on the defense of the cell against viruses (43–45). While in mice, saturation of the RNAi machinery was found to cause lethality (46), this has not yet been reported in arthropods (39). Furthermore, stimulation of the immune response upon dsRNA exposure is also a possibility. The receptors of the innate immune system can recognize dsRNA, in a sequence-independent manner, as a pathogenic signature leading to stimulation of the immune response (5). A study in honeybees, revealed an upregulation for the expression of several immune related genes, e.g., dsRNases, after feeding on non-specific dsRNA (43, 47). A similar link between dsRNA and immune pathways has been reported in bumblebees and silkworms (44, 48). While this immunostimulation can influence insect performance (49), no fundamental studies have been conducted exploring the likelihood of these potential adverse effects or to fully elucidate the molecular mechanisms behind these phenomena (45).

Important to take into consideration for the risk assessment is that, in contrast to conventional pesticides, RNAi-based products might take a longer time to display efficacy. This lag-effect should be considered when assessing RNAi-based products by extending the observation period and to take into account non-lethal phenotypes and life cycle analysis in addition to mortality (7, 16).

Despite the first SIGS-based products moving to the market, there is not yet a consensus on the data requirements needed for the risk assessment. In Europe, non-target testing for chemical pesticides requires, at the initial stage, a worst-case exposure analysis on two species which are highly sensitive to most classes of pesticides: the predatory mite *Typhlodromus pyri* and the parasitic wasp *Aphidius rhopalosiphi* (50). Only when adverse effects are observed in these species, additional assays with other beneficial species are required. Based on biological relevance (i.e., presence in the field), phylogenetic and functional diversity and the availability of reliable test protocols, the set of additional insects is composed of *Orius laevigatus* (Hemiptera), *Chrysoperla carnea* (Neuroptera), *Coccinella septempunctata* (Coleoptera), and *Aleochara bilineata* (Coleoptera) (50). In addition to these predators and parasitoids, tests on honey bees (*Apis mellifera*) and soil organisms, *Folsomia candida* (Collembola) or *Hypoaspis aculeifer* (Acari), are required if exposure of these is anticipated (51). However, while this set of organisms has been selected for chemical pesticides, it may not be suitable to assess non-target effects for all types of topically applied dsRNA-based products. Like risk assessment of GM plants, the most appropriate non-target species should be selected on a case-to-case basis. Therefore, three main criteria have been proposed: sensitivity, the organism must be sensitive to RNAi; relevance, the organism must be representative for valued taxa or functional groups likely to be exposed in the field; and availability and reliability, organisms must be available in sufficient quantity and quality at the appropriate life-stage and validated test protocols should be available (7). Table 1 provides an overview of model non-target species, including beneficial insects, soil and aquatic organisms for which validated protocols are available that allow the risk assessment of dsRNAs.

**Table 1:** Overview of RNAi experiments in potential non-target species.

Pollinators		*Bombus terrestris*	Taning et al. (52)
		*Apis mellifera*	Vélez et al. (53)
			US EPA (54)
			Bachman et al. (55)
			Flenniken and Andino (47)
		*Danaus plexippus*	Pan et al. (56)
Natural enemies	Parasitic wasps	*Pediobius foveolatus*	US EPA (57)
			Bachman et al. (55)
	Predators	*Coleomegilla maculata*	US EPA (57) Bachman et al. (55)
		*Orius insidiosus*	
		*Poecilus chalcites*	
		*Chrysoperla carnea*	
		*Aleochara bilineata*	
		*Coccinella septempunctata*	Jung et al. (58)
			Haller et al. (42)
		*Adalia bipunctata*	Haller et al. (42)
		*Nesidiocoris tenuis*	Sarmah et al. (59)
Soil fauna	Collembola	*Folsomia candida*	US EPA (57)
			Bachman et al. (55)
			Noland (60)
		*Sinella curviseta*	Pan et al. (61)
	Annelids	*Eisenia andrei*	US EPA (54, 57)
			Bachman et al. (55)
Aquatic organisms	Branchiopoda	*Daphnia pulex*	Schumpert et al. (62)
		*Artemia franciscana*	Dung et al. (63)
	Algae	*Chlamydomonas reinhardtii*	Kim and Cerutti (64)
		*Volvox carteri*	Cheng et al. (65)
		*Dunaliella salina*	Sun et al. (66)
			Jia et al. (67)
		*Phaeodactylum tricornutum*	De Riso (68)
		*Vaucheria frigida*	Takahashi et al. (69)
		*Euglena gracilis*	Iseki et al. (70)
	Fish	*Ictalurus punctatus*	US EPA (57)
			Bachman et al. (55)
Other organisms	Birds	*Gallus domesticus*	US EPA (57) Bachman (55)
		*Colinus virginianus*	

## Conclusion

The environmental (particularly spray) application of dsRNA (and their formulations) for RNAi-based pest control has a huge potential to replace detrimental traditional chemical pesticides, with species specific, sustainable, and environmentally friendly products. Field trials are confirming the power of these SIGS-based products and consequently promoting their transition to the market. However, being novel active compounds, current regulatory structures are challenged to provide a standardized legal framework for these dsRNA-based products. The legal framework for SIGS-based products in Australia can be an inspiration to create a specific niche for non-GMO RNAi-products in Europe. However, more pressing is the need for a well-defined risk assessment procedure. This analysis should comprise not only the dsRNA as active ingredient but also take in account the effect of formulations. Therefore, a thorough discussion is needed on regulatory and biosafety issues to ensure that the risk of these products is adequately assessed and a risk assessment and management framework is elaborated.

## Author Contributions

KDS drafted the manuscript and all authors (CT, LV, EV, PD, and GS) contributed to revising the manuscript. All authors approved the final version of the manuscript.

## Funding

LV is recipient of a doctoral fellowship from the Research Foundation Flanders (FWO) (grant number 1SA2720N) and CT is recipient of a senior postdoctoral fellowship from the Research Foundation Flanders (FWO) (grant number 12V5722N). This work is supported by the Special Research Fund of the Ghent University (Belgium) (grant number BOF22/GOA/010).

## Conflict of Interest

The authors declare that the research was conducted in the absence of any commercial or financial relationships that could be construed as a potential conflict of interest.

## Publisher's Note

All claims expressed in this article are solely those of the authors and do not necessarily represent those of their affiliated organizations, or those of the publisher, the editors and the reviewers. Any product that may be evaluated in this article, or claim that may be made by its manufacturer, is not guaranteed or endorsed by the publisher.
